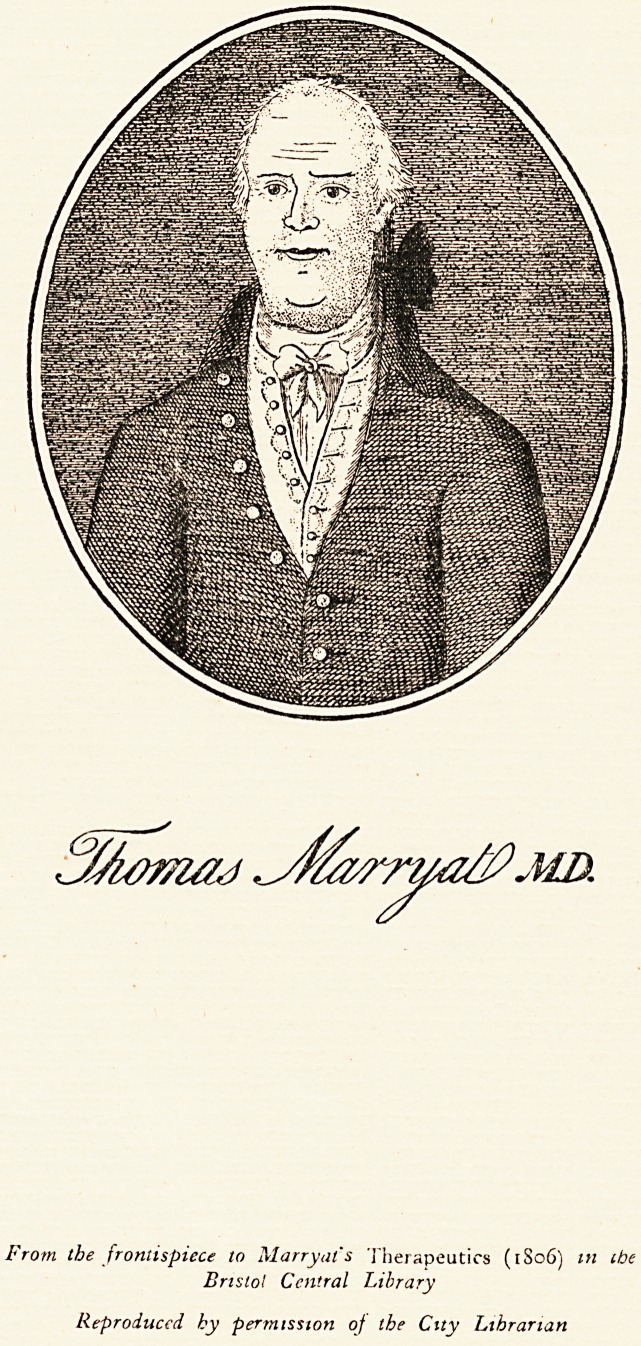# Thomas Marryat, M.D.: A Memoir

**Published:** 1935

**Authors:** A. S. MacNalty


### THOMAS MARRYAT, M.D.: A MEMOIR.

BY

A. S. MacNalty, M.D., F.R.C.P.,

---

Thomas Marryat was born in London in the year 1730. He had a retentive memory and a great love for letters from an early age. He read and spoke Latin with ease, and it is said that he could read any Greek author, even Lycophron, at nine years old. Coming of a long line of Puritan ancestry, he was intended for the dissenting ministry, but literature, the muses and the pleasures of the town had greater attractions for him than theology. *The Dictionary of National Biography* states that Marryat from 1747 to 1749 was a member of a poetical club which met at the Robin Hotel, Butcher Row, Strand, every Wednesday at 5 p.m., and seldom broke up until 5 a.m. in the next morning. Dr. Richard Brookes, Moses Browne, Stephen Duck the yokel poet, Martin Madan and Thomas Madox belonged to this club. Each member at the weekly meeting brought a poem and subjected it to the criticism of his fellows. The club's literary effusions supplied the wants of *The Gentleman's Magazine* and other periodicals.

Marryat had a lively although coarse wit. He was nicknamed by Dr. Brookes "Salvolatile," and was never known to laugh at his own jokes.

It soon became apparent that Marryat had no bent for the ministry. He became a medical student in Edinburgh and graduated M.D. His Bohemian habits clung to him and probably militated against his success in practice. In 1762 he made a tour of the continental medical schools, and afterwards visited America. In 1766 he settled in Antrim and the northern parts of Ireland, "where he was almost deified by the populace, and much esteemed by many persons of high rank and extensive patronage."

A great believer in drugs, he devoted two hours a day to non-paying patients, to whom he gave enormous experimental doses of drastic medicines.

For colic he drew off six or seven ounces of blood and prescribed :—

Colocynth, a scruple.
Purified opium, ten grains.
Vitriolated quicksilver,
Precipitated sulphur of antimony, of each five grains.
Simple syrup, enough for pills Eight ; two every hour till the pain ceases.

For dysentery he gave white paper cut into slips and boiled in milk. "N.B. — This never deceived me." Marryat was evidently no homœopath. In his preface to his book on therapeutics he writes:—

> "The large doses of *volatiles* and *narcotics* to be met with in these pages, may startle the reader. It is to be feared they are often trifled with by an inexcusable temerity. It is a certain fact, that small doses, at different times, have often no good effect, and that the opportunity of saving our patient, which a large dose at first, might have effected, is irretrievably lost. An extensive experience has convinced me, that many lives are to be preserved by an happy temerity."

In spite of his popularity Marryat wearied of Ireland, and in 1774 returned to England and resided at Shrewsbury. In 1785 he migrated to Bristol and delivered a course of lectures on therapeutics which was well attended. He acquired a reputation for restoring to health patients who had been given up by other doctors, and for a time kept his carriage and did well in practice. It is to be feared he was somewhat of an advertiser, for in order to excite notice he published a book called *The Philosophy of Masons*, which was so heterodox in opinions and licentious in language that even his best friends censured him for it.

The doctor's professional success waned. He was improvident and a *bon vivant* and saved nothing for the future. In his poverty he declined for himself all assistance from his well-to-do relations. At some stage or other of his wandering existence Marryat had married a wife. By now his family must have been educated and launched on the world, for we know of one son, Joseph Marryat, M.P., who was Chairman for the Committee at Lloyd's and colonial agent for the Island of Grenada, a substantial man who refused a baronetcy, and was honoured by an elegy from Campbell. He was not allowed to assist his father, who preferred to fix a paper upon the window of the Bush Coffee-Room, Bristol, inquiring "if anyone remembered there was such a person as Thomas Marryat ?" and informing passers-by that he "still lived or rather existed in Horfield Road."

In his last illness Marryat said he was not afraid to die, for he was no atheist, as the world supposed him to be. He died on the 29th of May, 1792, little more than a month before the birth of his distinguished grandson, the future Captain Frederick Marryat, who resembled him in his facility for literary composition, a tendency to be at odds with orthodoxy, eccentricity and hot temper. He also possessed a good deal of his grandfather's wit and humour.

Thomas Marryat was buried in the ground belonging to the Chapel in Lewin's Mead, Bristol. As for his personal characteristics in life, the author of a short Memoir says :—

*Thomas Marryat* M.D.

*From the frontispiece to Marryat's* Therapeutics (1806) *in the Bristol Central Library*

*Reproduced by permission of the City Librarian*

"The countenance of the Doctor was far from engaging, and, like Gibbon the Historian, he was unable to boast of the charms of his person. In his disposition, he was, latterly, morose ; with a bluntness in his manners, bordering upon perfect rudeness, aping the manners, or rather the *ill* manners of our lexicographer, Johnson. Notwithstanding this, he was a pleasant companion when he began to expand himself, but a perfect hedge-hog to strangers and those whom he disliked."

He was a man of inflexible integrity, and under his bearish exterior had a heart of genuine kindness, particularly for the poor and needy. He occupies a lower place in medicine than his learning and talents might have secured. His behaviour in practice at Bristol smacked too much of the empiric to render him acceptable to his professional brethren, and he was one of those people who chafe at conventional restrictions, tilt at windmills and irritate those in authority.

The best-known of Marryat's medical works is *Therapeutics or a New Practice of Physic,* "humbly inscribed to everybody," which was first published in Latin in 1758. This was reprinted in Dublin in 1764. A pocket edition entitled *The Art of Healing* attained great popularity. The twentieth edition was published at Bristol in 1805.

In 1756 or 1757 Marryat had earlier published at Ipswich *Medical Aphorisms, or a Compendium of Physic, founded on irrefragible principles,* much of which he afterwards retracted. He also wrote verse.

A new edition of his *Sentimental Fables for the Ladies* appeared in Bristol in 1791, republished from an Irish copy; of these it may be said that "some are good, some bad, some indifferent." They were well reviewed at the time, have the merit of being moral, were dedicated to Miss Hannah More and a large impression sold well.

## Figures and Tables

**Figure f1:**